# Long-term (5 year) safety of bronchial thermoplasty: Asthma Intervention Research (AIR) trial

**DOI:** 10.1186/1471-2466-11-8

**Published:** 2011-02-11

**Authors:** Neil C Thomson, Adalberto S Rubin, Robert M Niven, Paul A Corris, Hans Christian Siersted, Ronald Olivenstein, Ian D Pavord, David McCormack, Michel Laviolette, Narinder S Shargill, Gerard Cox

**Affiliations:** 1 Gartnavel General Hospital, University of Glasgow, Glasgow, UK; 2 Irmandade Santa Casa de Misericórdia da Porto Alegre, Brazil; 3 University Hospital of South Manchester and University of Manchester, Manchester, UK; 4 Department of Respiratory Medicine, Freeman Hospital, Newcastle University, Newcastle, UK; 5 Odense University Hospital, Odense, Denmark; 6 Montreal Chest Institute, McGill University, Montreal, Canada; 7 Glenfield General Hospital, University Hospitals of Leicester NHS Trust, Leicester, UK; 8 London Health Sciences Center, Ontario, Canada; 9 Laval Hospital, Laval University, Quebec, Canada; 10 Asthmatx, Inc., Sunnyvale, CA, US; 11 St. Joseph's Healthcare, McMaster University, Hamilton, Canada

## Abstract

**Background:**

Bronchial thermoplasty (BT) is a bronchoscopic procedure that improves asthma control by reducing excess airway smooth muscle. Treated patients have been followed out to 5 years to evaluate long-term safety of this procedure.

**Methods:**

Patients enrolled in the Asthma Intervention Research Trial were on inhaled corticosteroids ≥200 μg beclomethasone or equivalent + long-acting-beta_2_-agonists and demonstrated worsening of asthma on long-acting-β_2_-agonist withdrawal. Following initial evaluation at 1 year, subjects were invited to participate in a 4 year safety study. Adverse events (AEs) and spirometry data were used to assess long-term safety out to 5 years post-BT.

**Results:**

45 of 52 treated and 24 of 49 control group subjects participated in long-term follow-up of 5 years and 3 years respectively. The rate of respiratory adverse events (AEs/subject) was stable in years 2 to 5 following BT (1.2, 1.3, 1.2, and 1.1, respectively,). There was no increase in hospitalizations or emergency room visits for respiratory symptoms in Years 2, 3, 4, and 5 compared to Year 1. The FVC and FEV_1 _values showed no deterioration over the 5 year period in the BT group. Similar results were obtained for the Control group.

**Conclusions:**

The absence of clinical complications (based on AE reporting) and the maintenance of stable lung function (no deterioration of FVC and FEV_1_) over a 5-year period post-BT in this group of patients with moderate to severe asthma support the long-term safety of the procedure out to 5 years.

## Background

Asthma continues to be a major health concern worldwide, with over 23 million people in the United States who suffer with this disease [1]. Approximately 5-10% of these patients are characterized as having severe persistent asthma based on continued presence of asthma symptoms despite treatment with current state-of-the-art medications [2]. Poorly controlled asthma impacts the patient's quality of life, increases healthcare utilization, and imposes both a social as well as an economic burden [3-8].

The recent approval of the Alair^® ^Bronchial Thermoplasty System for delivering bronchial thermoplasty (BT) provides an additional option for managing patients with severe asthma. BT provides therapeutic benefit by reducing the amount of excess smooth muscle in the airways, with the resultant effect of reducing bronchoconstriction in response to asthma triggers. Results from the Asthma Intervention Research (AIR) Trial, the first randomized clinical trial of BT which compared BT plus standard-of-care therapy (inhaled corticosteroids (ICS) and long-acting-β_2_-agonists (LABA)) to standard-of-care alone, demonstrated that the mean rate of mild exacerbations, as compared with baseline, was reduced in the BT group but was unchanged in the control group. Furthermore at 12 months, when subjects were on ICS alone, there were significantly greater improvements in the BT group than in the control group in the morning peak expiratory flow, scores on the AQLQ and ACQ, the percentage of symptom-free days and symptom scores, while fewer puffs of rescue medication were required [9]. Two additional randomized controlled trials, the Research in Severe Asthma (RISA) Trial [10], and the sham-controlled Asthma Intervention Research 2 (AIR2) Trial [11], have provided additional support for the effectiveness of BT. The safety of BT over the post-treatment period out to one year was established in all 3 randomized clinical studies. Because BT is a novel treatment that alters the amount of airway smooth muscle, and asthma is a chronic disease that is associated with structural changes in the airway wall, it is important to know whether this treatment is associated with any longer term adverse outcomes. Longer-term safety data out to 5 years are available for a small cohort of BT treated subjects from the first clinical study of patients with mild to moderate asthma [12,13]. We now describe the safety profile of BT out to 5 years post-treatment from patients with moderate to severe asthma from the AIR Trial [9].

## Methods

### Study subjects

All patients completing the 12-month follow-up evaluations in the AIR Trial (BT group: standard-of-care + BT; Control group: standard-of-care) were invited to participate in a 5 year post-treatment extension study to evaluate longer-term safety under a new protocol (NCT00448812). The exclusion criteria for participation in the extension study were: participation in another clinical trial involving respiratory intervention, or new diagnosis of psychiatric disorder which in the judgment of the investigator could interfere with provision of informed consent, completion of tests, therapy, or follow-up.

The key inclusion criteria to establish eligibility of patients to participate in the original AIR Trial were: 18-65 year old ambulatory adults; stable asthma (no unscheduled visits or change to medications within 6 weeks prior to randomization); requiring inhaled corticosteroids (ICS): at least 200 μg beclomethasone or equivalent per day, and long-acting β_2_-agonist (LABA): at least 100 μg salmeterol or equivalent per day; demonstration of worsening of asthma after 2-week LABA withdrawal; pre-bronchodilator FEV_1 _≥ 60% and ≤ 85% predicted; methacholine PC_20 _< 8 mg/ml; and, non-smoker x 1 yr; if former smoker, less than 10 pack-year history. The key exclusion criteria were: history of ≥ 3 lower respiratory tract infections per year (requiring antibiotics); and, requirement of > 4 puffs/day of a short-acting β_2_-agonist, excluding for exercise. The protocol for the longer-term extension study was approved by the respective Institutional Review Boards/Ethics Committees at each participating institution prior to obtaining a signed informed consent from the study participants.

### Study procedures

Subjects in the BT group were evaluated annually; at Year 2, Year 3, Year 4, and Year 5 after their last treatment bronchoscopy. Subjects in the Control group were evaluated at Year 2 and Year 3 and then exited from the study. Year 1 data are provided for matched pairs in both the BT and Control groups comprising those subjects that enrolled in the longer-term follow-up.

Annual evaluations included a physical examination, pre- and post-bronchodilator spirometry, static lung volumes, diffusing capacity, chest x-ray (PA and Lateral), methacholine PC_20 _(out to Year 3 only), as well as active solicitation of information on any adverse events, emergency room visits and hospitalizations for asthma symptoms, oral corticosteroid pulses for worsening asthma symptoms, and any changes in maintenance asthma medications. Maintenance asthma medication use, oral corticosteroid use, adverse events, emergency room visits and hospitalizations were verified through medical record review for about 80% of the subjects whose primary care was under the supervision of the investigator at their respective institution. X-ray observations reported by radiologists at each site were collectively reviewed by an independent pulmonologist to assess clinical relevance of the observations if any.

During Year 1, adverse events were solicited during 12 office visits and 9 telephone contacts over the course of the year, as well as a review of the medical chart. During the longer-term follow-up, adverse events were actively solicited from the subject during the annual evaluation and through a review of the medical chart for the prior year for subjects managed at the investigator's institute. The recording of adverse events in Year 1 (AIR Trial) differed from the subsequent years (AIR Extension Study) in that in Year 1, multiple symptoms associated with an adverse event were collected as individual adverse events; while in the subsequent years, an adverse event with multiple symptoms was counted as a single adverse event e.g., multiple respiratory symptoms associated with worsening of asthma were considered as a single event called "asthma (multiple symptoms)" adverse event.

### Statistical Analyses

#### Demographics

Group means were compared using Student's t-test. *Hospitalizations and Emergency Room Visits for respiratory symptoms*: The respective number of subjects completing each annual follow-up visit was used to calculate the proportion of subjects with hospitalizations or emergency room visits for respiratory symptoms in each year. Fisher's Exact test was used to compare proportion of subjects with respiratory hospitalizations and emergency room visits in the BT and Control groups during Years 1, 2 and 3. Trends in the percent of subjects with hospitalizations or emergency room visits for respiratory symptoms across Years 1 to 5 were investigated using a repeated measures logistic regression (generalized estimating equation), modeling the percent of subjects reporting the event. *Maintenance medications ICS dose*: Change from Baseline to each follow-up year in ICS dose was analyzed with a Signed Rank test.

## Results

### Demographics and Clinical Characteristics

Forty five (45) of the 52 subjects in the BT group (87%), and 24 of the 49 subjects in the Control group (49%) who completed the Year 1 evaluation opted to participate in the extension study. The 7 subjects in the BT group and 25 subjects in the Control group who declined to participate in the long-term follow-up did so for personal reasons, and not due to mortality. The numbers of subjects enrolling for the longer-term follow-up and those completing scheduled annual evaluations are summarized in Table 1.

**Table 1:** Number of Subjects Evaluated Annually out to 5 Years

	Subjects Completing 1 Year Follow-up in AIR Trial	Subjects Enrolling for Longer-Term Follow-up	Subjects Completing Follow-up			
			
			Year 2	Year 3	Year 4	Year 5
BT	52	45	41^a^	41^b^	43	42^c^
Control	49	24	23^d^	21^e^	-	-

a: 4 subjects missed visit.

b: 2 subjects missed visit; 1 subject withdrew consent; 1 subject Lost-to-Follow-up.

c: 1 subject missed visit.

d: 1 subject missed visit.

e: 2 subjects withdrew consent after completing Year 2 evaluation, and 1 subject withdrew consent mid-Year 3.

Control group subjects exited from study after completing Year 3 evaluation.

The baseline demographic information and clinical characteristics (at time of entry into the AIR Trial) for subjects participating in the extension study are provided in Table 2. The groups were well matched with no statistically significant differences between them for any given parameter.

**Table 2:** Baseline Demographics and Clinical Characteristics

	BT (n = 45)	Control (n = 24)
Age (yrs)	40.0 ± 11.2	40.8 ± 12.1

Gender	Male 19 (42%)	Male 9 (38%)
	Female 26 (58%)	Female 15 (63%)

Race	White 41 (91%)	White 22 (92%)
	Black 3 (7%)	Black 2 (8%)
	Asian 1 (2%)	Asian 0 (0%)

Height (cm)	166.1 ± 9.6	164.8 ± 7.7

Weight (kg)	76.3 ± 23.3	77.7 ± 16.9

Inhaled Corticosteroid Dose (μg)^a^	1305 ± 880	1141 ± 1053

LABA Dose (μg)^b^	109 ± 34	100 ± 15

Symptom-Free Days (%)	33.3 ± 34.3	46.1 ± 41.0

Asthma Control Questionnaire (ACQ) Score	1.3 ± 0.6	1.2 ± 0.7

Asthma Quality of Life Questionnaire (AQLQ) Score	5.6 ± 0.9	5.6 ± 0.9

Rescue Medication Use (No. of puffs/7days)	10.6 ± 14.7	5.5 ± 10.4

Emergency Room Visits for Respiratory Symptoms in prior 12 months ^c^ No. Events (No. Subjects)	3 (3)	0 (0)

Hospitalizations for Respiratory Symptoms in prior 12 months ^c^ No. Events (No. Subjects)	3 (3)	2 (2)

**Lung Function Measures**		

Morning PEF (L/min)	368.4 ± 99.7	394.1 ± 111.7

Pre-Bronchodilator FEV_1 _(% predicted)	72.5 ± 10.9	74.9 ± 8.9

Post-Bronchodilator FEV_1 _(% predicted)	84.4 ± 13.8	86.1 ± 9.5

Diffusion Capacity (mL/min/mm Hg)	15.7 ± 10.7	15.9 ± 11.7

Total Lung Capacity (L)	6.0 ± 1.2	5.9 ± 1.3

Residual Volume (L)	2.1 ± 0.7	2.0 ± 0.7

Methacholine PC_20 _(mg/ml)	0.25	0.28
Geometric mean (range)	(0.2, 0.4)	(0.1, 0.6)

*Definition of abbreviations: *BT = Bronchial Thermoplasty; LABA = Long-Acting β_2_-Agonist;

PEF = Peak Expiratory Flow Rate; FEV_1 _= Forced Expiratory Volume in 1 second.

Values are mean ± SD.

a: Beclomethasone or equivalent.

b: Salmeterol or equivalent.

c: Patient reported.

BT versus Control: All parameters, Not significant (Student's t-test of the mean).

### Safety

#### Adverse Events

There were no incidences of pneumothorax, intubation, mechanical ventilation, cardiac arrhythmias, or death as a result of BT treatment over the 5 year follow-up.

Respiratory adverse events reported during the course of the 5 year follow-up are summarized in Table 3. During Year 1, the rate of respiratory adverse events in both the BT and Control groups was higher as a result of the method of recording the adverse events whereby multiple symptoms associated with an adverse event were recorded as separate adverse events. In subsequent years (Year 2 to Year 5), an adverse event with multiple symptoms was recorded as a single adverse event. The rate of respiratory adverse events in the BT group (AEs/subject) remained stable in years 2 to 5 following BT. A repeated measures analysis for the "asthma (multiple symptoms)" adverse events which reflects worsening asthma control showed no deterioration over time from Year 2 to Year 5 (p = 0.47). During Year 2 and Year 3, when data for the Control group was collected, the respiratory adverse event rate between the BT and Control groups was not significantly different.

**Table 3:** Summary of Respiratory Adverse Events

		Year 1	Year 2	Year 3	Year 4	Year 5
	
Number of subjects	BT	45 ^a^	45	43	43	42
	
	Control	24^a^	24	21	-- ^b^	-- ^b^
Number of subjects reporting (Percent of subjects)	BT	38 (84%)	24 (53%)	24 (56%)	23 (53%)	22 (52%)
	
	Control	18 (75%)	13 (54%)	12 (57%)	--	--

Events per subject	BT	4.5	1.2	1.3	1.2	1.1
	
	Control	3.1	1.2	1.3	--	--

a: Year 1 data only for subjects who enrolled for longer-term follow-up.

b: Control group subjects exited from Study after Year 3 evaluations.

Respiratory adverse events occurring at a by-subject incidence rate of ≥3.0% in any of the years are given in Table 4. For the majority of respiratory adverse events, the incidence rates were stable during each year from Year 2 to Year 5 in the BT group, and from Year 2 and Year 3 in the Control group. The respiratory adverse events were typical of asthma. One subject in the BT group who had undergone the procedure was diagnosed with a lung abscess in the previously treated left upper lobe at 14 months (Year 2), and was resolved with surgical resection. The subject had undergone BT uneventfully and had completed the 12 month follow-up with normal spirometric values and good asthma control (Post-BD FEV_1 _at baseline was 2.27L, and at 12 months was 2.33L). Histological examination of the dissected lung did not reveal an obstruction or any other potentially contributory abnormality in the airways as a result of the treatment. The abscess was considered secondary to an infection. At the time of exit from the study at 5 years, the post-BD FEV_1 _for this subject was 1.78L compared to baseline value of 2.27L.

**Table 4:** Subjects with Respiratory Adverse Events (All events reported at ≥3.0% in any year)

	**Year 1 **^**a**^		Year 2		Year 3		**Year 4 **^**b**^	**Year 5 **^**b**^
**Adverse Event**	**BT** **(n = 45)**	**Control** **(n = 24)**	**BT** **(n = 45)**	**Control** **(n = 24)**	**BT** **(n = 43)**	**Control** **(n = 21)**	**BT** **(n = 43)**	**BT** **(n = 42)**

Dyspnea	19 (42.2%)	12 (50.0%)	4 (8.9%)	3 (12.5%)	4 (9.3%)	3 (14.3%)	4 (9.3%)	4 (9.5%)
Cough	17 (37.8%)	7 (29.2%)	4 (8.9%)	1 (4.2%)	2 (4.7%)	3 (14.3%)	3 (7.0%)	2 (4.8%)
Wheeze	14 (31.1%)	4 (16.7%)	2 (4.4%)	1 (4.2%)	3 (7.0%)	1 (4.8%)	3 (7.0%)	2 (4.8%)
Nasal congestion	13 (28.9%)	5 (20.8%)	2 (4.4%)	0	0	0	0	1 (2.4%)
Upper Respiratory Tract Infection	10 (22.2%)	2 (8.3%)	11 (24.4%)	4 (16.7%)	8 (18.6%)	4 (19.1%)	8 (18.6%)	4 (9.5%)
Productive cough	9 (20.0%)	5 (20.8%)	2 (4.4%)	1 (4.2%)	2 (4.7%)	0	0	1 (2.4%)
Chest discomfort	8 (17.8%)	3 (12.5%)	2 (4.4%)	2 (8.3%)	3 (7.0%)	1 (4.8%)	1 (2.3%)	2 (4.8%)
Nasopharyngitis	6 (13.3%)	0	1 (2.2%)	0	0	0	1 (2.3%)	1 (2.4%)
Nocturnal Dyspnea	6 (13.3%)	2 (8.3%)	0	0	0	0	0	0
Respiratory Tract Infection ^c^	5 (11.1%)	5 (20.8%)	3 (6.7%)	2 (8.3%)	5 (11.6%)	1 (4.8%)	5 (11.6%)	4 (9.5%)
Pharyngolaryngeal pain	5 (11.1%)	3 (12.5%)	0	0	0	0	0	0
Respiratory Tract congestion	4 (8.9%)	2 (8.3%)	0	0	0	0	0	0
Discolored sputum	4 (8.9%)	0	3 (6.7%)	0	0	0	0	0
Rhinitis	2 (4.4%)	0	0	0	1 (2.3%)	0	0	2 (4.8%)
Bronchitis ^d^	1 (2.2%)	0	1 (2.2%)	1 (4.2%)	1 (2.3%)	2 (9.5%)	1 (2.3%)	1 (2.4%)
Pharyngitis	1 (2.2%)	1 (4.2%)	0	0	0	0	0	0
Pleuritic Pain	1 (2.2%)	1 (4.2%)	0	0	0	0	0	0
Rhinorrhea	1 (2.2%)	1 (4.2%)	0	0	1 (2.3%)	0	0	0
Asthma (multiple symptoms) ^e^	0	0	4 (8.9%)	2 (8.3%)	7 (16.3%)	1 (4.8%)	7 (16.3%)	6 (14.3%)
Sinusitis	0	0	1 (2.2%)	1 (4.2%)	2 (4.7%)	0	2 (4.7%)	2 (4.8%)
Nasal polyps	0	0	1 (2.2%)	0	0	0	2 (4.7%)	0
Pneumonia	0	0	0	0	1 (2.3%)	1 (4.8%)	0	0

a: Year 1 data only for subjects who enrolled for longer-term follow-up. Adverse events solicited from patient during multiple office visits in Year 1. In subsequent years, adverse events solicited only at annual follow-up visit.

b: Control group subjects exited from Study after Year 3 evaluations.

c: Includes adverse events reported as "Respiratory Tract Infection" and "Lower Respiratory Tract Infection".

d: Includes adverse events reported as "Bronchitis" and "Tracheobronchitis".

e: Asthma - In Year 1, all symptoms were collected as individual adverse events; in subsequent years, multiple symptoms of asthma exacerbation were considered as a single adverse event.

#### Healthcare Utilization Events

During Year 1 and Year 2, more subjects in the BT group required hospitalizations for respiratory symptoms than the Control group, but these differences were not significant. There was one hospitalization for respiratory symptoms in the Control group in Year 3. Over the course of the 5 year post-BT follow-up, the number of hospitalizations, and the proportion of subjects experiencing hospitalizations for respiratory symptoms did not get worse compared to Year 1 after BT (Table 5) (p = 0.16; repeated measures analysis for proportion of subjects). Similarly, the number of emergency room (ER) visits for respiratory symptoms and the proportion of subjects experiencing ER visits for respiratory symptoms remained comparable and did not get worse in Years 2, 3, 4, and 5 compared to Year 1 (Table 5) (p = 0.55; repeated measures analysis). The number of ER visits for respiratory symptoms in the Control group in Years 2 and 3 were comparable to the BT group (p = 0.41 and p = 1.00, respectively; Fisher's Exact test).

**Table 5:** Summary of Healthcare Utilization Events

		Hospitalizations Percent of Subjects [95% CI] (Number of Events)			Emergency Room Visits Percent of Subjects [95% CI] (Number of Events)		
	**Total Number of Subjects**	**BT**	**Control**	**p-value**^**b**^	**BT**	**Control**	**p-value**^**b**^

Year 1	BT = 45 Control = 24	6.7% [0.0, 14.0] (3)	0	0.55	4.4% [0.0, 10.5] (2)	0	0.54
Year 2	BT = 45 Control = 24	6.7% [0.0, 14.0] (3)	0	0.55	6.7% [0.0, 14.0] (3)	12.5% [0.0, 25.7] (3)	0.41
Year 3	BT = 43 Control = 21	2.3% [0.0, 6.8] (3)	4.8% [0.0, 13.9] (1)	1.00	4.7% [0.0, 10.9] (3)	4.8% [0.0, 13.9] (3)	1.00
Year 4 ^a^	BT = 43 Control = 0	2.3% [0.0, 6.8] (1)	--		9.3% [0.6, 18.0] (6)	--	
Year 5 ^a^	BT = 42 Control = 0	2.4% [0.0, 7.0] (1)	--		4.8% [0.0, 11.2] (2)	--	

a: Control group subjects exited from Study after Year 3 evaluations.

b: p-value from Fisher's Exact test.

#### Oral Corticosteroid Use for Asthma Symptoms

The frequency of OCS usage for worsening of asthma symptoms is shown in Table 6. Neither the rate of OCS usage nor the proportion of subjects requiring OCS pulses showed any worsening over the 5 year period in the BT group. OCS usage for asthma symptoms was comparable between the BT and Control groups during Years 1, 2 and 3.

**Table 6:** Use of Oral Corticosteroid Pulses for Asthma Symptoms

		Oral Corticosteroid Pulses: Events/Subject/Year (Percent of Subjects)	
		
	Total Number of Subjects	BT	Control
Year 1 ^a^	BT = 45 Control = 24	0.60 (24.5%) ^c^	0.42 (20.8%) ^c^
Year 2	BT = 45 Control = 24	0.49 (24.5%)	0.54 (33.3%)
Year 3	BT = 43 Control = 21	0.33 (25.6%)	0.52 (23.8%)
Year 4 ^b^	BT = 43 Control = 0	0.63 (27.9%)	--
Year 5 ^b^	BT = 42 Control = 0	0.62 (30.9%)	--

a: Year 1 data only for subjects who enrolled for longer-term follow-up.

b: Control group subjects exited from Study after Year 3 evaluations.

c: Includes adverse events reported as "Asthma", "Exacerbations of asthma", "Wheeze", "Dyspnea", and "Respiratory infection".

#### Maintenance Asthma Medication Use

During the review of maintenance asthma medications at each annual visit, the medication dosages for a number of subjects had been adjusted to reflect their current level of asthma control. Compared to their baseline pre-BT usage, over the course of the 5 years, an average of 57% of BT subjects reported a decrease in their LABA use, 40% of subjects reported no change in their LABA use, and 3% reported an increase in their LABA use. In the Control group, over the course of the 3 years, an average of 54% of Control subjects reported a decrease in their LABA use, 43% of subjects reported no change in their LABA use, and 3% reported an increase in their LABA use. In the same period an average of 49% of subjects in the BT group and 47% of subjects in the Control group were no longer taking LABA as controller medication.

The mean reduction from baseline in ICS dose for BT subjects was 182 μg/day (p = 0.09), 135 μg/day (p = 0.32), 150 μg/day (p = 0.25), 151 μg/day (p = 0.23), and 194 μg/day (p = 0.16) at Years 1, 2, 3, 4, and 5 respectively (p-values from a Signed Rank test). At Year 3, the mean reduction in the ICS daily dose in the Control group was 112 μg/day. The reduction in ICS was not significantly different between the BT group and the Control group at years 2 and 3 (p = 0.93 for Year 2, and p = 0.92 for Year 3), years when data were available for both the BT and Control groups. At 3 years the proportion of subjects in the BT group with changes from baseline in their maintenance ICS dose was 27% with a decrease, 56% with no change, and 17% with an increase. The corresponding numbers for the Control group were 29%, 52% and 19% respectively.

#### Review of Serial (Annual) Chest X-rays

18 of the 45 BT subjects (40%), and 9 of the 24 Control subjects (37%) had finding(s) noted on chest x-rays. Findings ranged from air trapping, pleural thickening, increased density/consolidation, hyperinflation, nodules/granuloma, increased vascular markings, and bronchial wall thickening. None of the findings noted in either group were clinically significant structural changes. The findings noted were either pre-existing, minor and transient, or consistent with acute inter-current illnesses that typically occur in patients with moderate and severe asthma.

#### Pulmonary Function Tests

**Figure 1:**
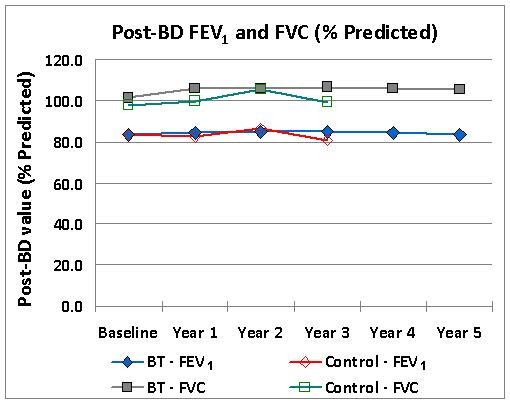
**FEV_1 _and FVC over Time**. Post-bronchodilator FEV_1 _and FVC over time. Data represent group mean values for each year.. Note that participation of the Control subjects in the study was terminated after Year 3 evaluation.

Pulmonary function tests were performed when subjects were taking only ICS as their maintenance asthma medication (either after a 2 week withdrawal of LABAs for subjects that were on ICS+LABA or for subjects on just ICS). The mean post-bronchodilator FEV_1 _and FVC values over time are presented graphically in Figure 1, respectively. Both measures of lung function (FEV_1 _and FVC) remained stable and showed no deterioration over the 5 year period post-BT.

Similarly, total lung capacity and residual volumes remained stable out to 5 years in the BT group. There were small improvements over baseline in Diffusion Capacity in both the BT and Control groups over the course of the study.

There was an improvement over baseline in the methacholine PC_20 _doubling in the BT group compared to the Control group in each year out to Year 3. The differences between groups were statistically significant in Year 2 and Year 3, but not in Year 1 (methacholine PC_20 _doublings BT versus Control: Year 1: 1.53 ± 2.29 vs 1.00 ± 2.46, p = 0.378; Year 2: 1.21 ± 2.99 vs -0.47 ± 2.31, p = 0.024; Year 3: 1.31 ± 2.96 vs -0.44 ± 2.27, p = 0.025).

## Discussion

The recently approved Alair System for bronchial thermoplasty provides a new treatment option for patients with severe asthma that remain symptomatic despite taking inhaled corticosteroids and long-acting-β_2_-agonists, the current standard-of-care medications. Three randomized clinical trials in patients with differing severity of asthma have demonstrated the safety of this device-based treatment out to one year [9-11]. In the AIR Trial, at 12 months post-treatment, while patients were on ICS alone, these benefits included significantly greater improvements in the BT group than in the Control group in mild exacerbation rates, morning peak expiratory flow, scores on the AQLQ and ACQ, the percentage of symptom-free days and symptom scores, while fewer puffs of rescue medication were required [9]. Each of these studies has also reported on the short-term (treatment period) and long-term (out to 12 months) adverse event profile associated with the bronchial thermoplasty. The reported increase in respiratory adverse events in the short-term represented the further aggravation of the worsening of asthma symptoms that is associated with bronchoscopy in patients with asthma [14]. During the long-term post-treatment period out to 12 months, fewer subjects in the BT group reported respiratory adverse events [9-11]. Data are now presented for the safety of this treatment over a 5 year period.

BT is a novel treatment that alters the amount of airway smooth muscle, and asthma is chronic disease that is associated with structural changes in the airway wall, it was important to know whether this treatment is associated with any longer term adverse outcomes. The participation of patients originally enrolled in the AIR Trial in this additional 4 year follow-up study provides data to support the longer-term safety of BT out to at least 5 years. The absence of serious events indicates that the integrity of the airways is not compromised over the long term as a result of BT treatment. This is supported by the observation of no deterioration of baseline FEV_1 _or FVC over this period, and the ability of the airways to respond to bronchodilator administration. While the rate of decline in lung function can vary in asthma, with some subjects demonstrating equivalent declines to those without airways disease, and others suffer an accelerated decline [15-17], it is reassuring that following BT, there was no significant loss of lung function over a 5 year period. The rate of occurrence of respiratory related adverse events remained low and stable between Year 2 and Year 5 (1.1 to 1.3 events/subject/year) and comparable to the Control group for Year 2 and Year 3 (1.2 and 1.3 events/subject/year, respectively). The frequency and method of collecting adverse events in this study was different in Year 1 compared to the other 4 years, resulting in a higher rate of respiratory adverse events in both the BT and Control groups in Year 1. During Year 1, the solicitation of adverse events was more frequent (12 office visits and 9 telephone contacts) compared to subsequent years when adverse events were solicited once a year and could be subject to recall bias. Also important is the fact that during Year 1, multiple symptoms for a given adverse event were recorded as individual adverse events as compared to the subsequent years when multiple related symptoms were recorded as a single event. Because of these methodological issues, comparisons of adverse event rates within each group for Year 1 and subsequent years are not appropriate. The lower rates of respiratory adverse events in Years 2 to 5 compared to Year 1 may reflect some underreporting as a result of recall bias at the yearly solicitation of adverse events. Every effort was made to carefully solicit adverse events from study subjects during annual evaluations and augmented with a thorough review of medical records at participating institutions. Review of medical charts is lacking for instances where a subject may have been treated for adverse event(s) at a different institution.

Measures such as ER Visits and hospitalizations for respiratory symptoms are generally considered to be important measures of safety, especially if an intervention results in an increase in the rate of one or more of these events. Consistent with the low rate of respiratory adverse events is the stable incidence of healthcare utilization events (hospitalizations and emergency room visits for respiratory symptoms). There was no worsening of the proportion of subjects in the BT group experiencing hospitalizations or ER visits for respiratory symptoms beyond Year 1 out to 5 years. The rates of ER visits and the proportion of subjects with ER visits in the Control group were comparable to the BT group during Years 1, 2, and 3. Longer-term safety of BT is supported further by our findings of no deterioration in measurements of static and dynamic lung volumes or diffusing capacity over the 5 years post-treatment.. In addition airway responsiveness to methacholine remained stable in the BT group out to the last measurement performed at year 3 post-treatment. Stable lung function measures in this group of patients with moderate to severe asthma support the previously reported conclusion based on lung function measures [12,13] and high resolution CT [13] that BT-treated airways do not develop late scarring that could result in narrowing of the airways [12].

Although x-rays are not sensitive enough for the purpose of demonstrating structural abnormalities, a review of the serial x-rays obtained annually did not reveal any clinically significant findings. While the use of high resolution computed tomography (HCRT) would have been more informative, it was not performed in this study.

Bronchial hyperresponsiveness to methacholine improved in the thermoplasty group in years 2 and 3, but not in year 1. This is an interesting result in favour of a long-term efficacy of the procedure. However, the lack of follow-up of the control group on years 4 and 5 and the observational nature of the data limits the relevance of this finding.

There are several limitations to the study. Firstly, not all patients enrolled in the Asthma Intervention Research Trial participated in long-term follow-up, although none of the reasons for non-participation was due to mortality. Secondly, the follow-up period was longer in patients who received BT (at 5 years) compared to the Control group (3 years). Subjects who had been randomized to the Control group in the predecessor AIR Trial may have opted to pursue other treatment options, and for those that agreed to the longer-term follow up, it was deemed to be unethical [18] to require them to withhold other alternative treatment options (including new experimental treatments) for a period of 5 years in order to avoid confounding of data. Nevertheless a high proportion of patients who received BT (86%) participated in long-term follow-up, which provides support for the generalizability of the safety data. Thirdly, the solicitation of adverse events on a yearly basis has the potential of under reporting due to recall bias. However, the occurrence of major events such as severe exacerbations (steroid pulses for asthma symptoms), emergency room visits, and hospitalizations is less frequent and less likely to be forgotten and therefore not reported. Medical charts were reviewed to verify steroid pulses prescribed for asthma symptoms (severe exacerbations) and reported adverse events or to identify adverse events that may not have been reported by the subject, unless the subject had presented at a different medical facility for an adverse event during that year. Finally, the study was not blinded and this may add bias to the eliciting of adverse events. Despite these limitations, over the 5 year follow-up of BT treated subjects, the absence of unexpected respiratory adverse events, no increases in hospitalizations or emergency room visits for respiratory symptoms, and maintenance of stable lung function demonstrated safety of BT out to 5 years.

## Conclusions

This report provides long-term safety data on BT in patients in whom BT was performed by trained operators, and patients were carefully observed especially in the first year. The absence of clinical complications based on adverse event reporting, healthcare utilization events, and the maintenance of stable lung function (no deterioration of FEV_1_) over a 5-year period post-BT in patients with moderate to severe asthma suggest long-term safety of the procedure out to 5 years.

## Abbreviations

AE: Adverse Event; ACQ: Asthma Control Questionnaire; AIR: Asthma Intervention Research; AIR2: Asthma Intervention Research 2; AQLQ: Asthma Quality of Life Questionnaire; BT: Bronchial thermoplasty; FEV_1_: Forced Expiratory Volume in 1 second; FVC: Forced Vital Capacity; HRCT: High resolution computed tomography; ICS: Inhaled corticosteroid; L: Liters; LABA: Long-Acting-β_2_-Agonist; OCS: Oral corticosteroid; PC_20_: Provocative Concentration causing 25% drop in FEV_1_; PEF: Peak Expiratory Flow; RISA: Research in Severe Asthma; SD: Standard deviation

## Competing interests

*Neil C. Thomson, Adalberto S. Rubin, Robert M. Niven, Paul A. Corris, Hans Christian Siersted, Ronald Olivenstein, Ian D. Pavord,, David McCormack, Michel Laviolette, Gerard Cox *all received industry-sponsored grant funding from Asthmatx, the manufacturers of the Alair^® ^System, for participating in clinical trials. *Narinder S Shargill *is an employee of Asthmatx.

## Authors' contributions

*NCT, M.D.*: Contributed to the acquisition and interpretation of the data, writing and revision of the manuscript, and gave final approval of the version to be published. Had full access to the data and will vouch for the integrity of data analysis, and the accuracy and completeness of the reported data.

*ASR, M.D.*: Contributed to the acquisition and interpretation of the data, and gave final approval of the version to be published.

*RMN, M.D.*: Contributed to the acquisition and interpretation of the data, writing and revision of the manuscript, and gave final approval of the version to be published.

*PAC, M.D.*: Contributed to the acquisition and interpretation of the data, and gave final approval of the version to be published.

*HCS, M.D.*: Contributed to the acquisition and interpretation of the data, and gave final approval of the version to be published.

*RO, M.D.*: Contributed to the acquisition and interpretation of the data, and gave final approval of the version to be published.

*IDP, M.D.*: Contributed to the acquisition and interpretation of the data, and gave final approval of the version to be published.

*DM, M.D.*: Contributed to the acquisition and interpretation of the data, and gave final approval of the version to be published.

*ML, M.D.*: Contributed to the acquisition and interpretation of the data, and gave final approval of the version to be published.

*NSS, PhD.*: Contributed to the execution of the study, acquisition and interpretation of the data, writing and revision of the manuscript, and gave final approval of the version to be published. Had full access to the data and will vouch for the integrity of data analysis, and the accuracy and completeness of the reported data.

*GC, M.B.*: Contributed to the acquisition and interpretation of the data, writing and revision of the manuscript, and gave final approval of the version to be published. Had full access to the data and will vouch for the integrity of data analysis, and the accuracy and completeness of the reported data.

## Pre-publication history

The pre-publication history for this paper can be accessed here:

http://www.biomedcentral.com/1471-2466/11/8/prepub
